# Thyroid and Corticosteroid Signaling in Amphibian Metamorphosis

**DOI:** 10.3390/cells11101595

**Published:** 2022-05-10

**Authors:** Bidisha Paul, Zachary R. Sterner, Daniel R. Buchholz, Yun-Bo Shi, Laurent M. Sachs

**Affiliations:** 1 Department of Biological Sciences, University of Cincinnati, Cincinnati, OH 45221, USA; paulbi@mail.uc.edu (B.P.); sternezr@mail.uc.edu (Z.R.S.); buchhodr@ucmail.uc.edu (D.R.B.); 2 Section on Molecular Morphogenesis, National Institute of Child Health and Human Development, National Institutes of Health, Bethesda, MD 20814, USA; shiyu@mail.nih.gov; 3 UMR 7221 Molecular Physiology and Adaption, CNRS, Museum National d’Histoire Naturelle, 57 Rue Cuvier, CEDEX 05, 75231 Paris, France

**Keywords:** thyroid hormones, corticosteroids, hormonal crosstalk, development, metamorphosis

## Abstract

In multicellular organisms, development is based in part on the integration of communication systems. Two neuroendocrine axes, the hypothalamic–pituitary–thyroid and the hypothalamic–pituitary–adrenal/interrenal axes, are central players in orchestrating body morphogenesis. In all vertebrates, the hypothalamic–pituitary–thyroid axis controls thyroid hormone production and release, whereas the hypothalamic–pituitary–adrenal/interrenal axis regulates the production and release of corticosteroids. One of the most salient effects of thyroid hormones and corticosteroids in post-embryonic developmental processes is their critical role in metamorphosis in anuran amphibians. Metamorphosis involves modifications to the morphological and biochemical characteristics of all larval tissues to enable the transition from one life stage to the next life stage that coincides with an ecological niche switch. This transition in amphibians is an example of a widespread phenomenon among vertebrates, where thyroid hormones and corticosteroids coordinate a post-embryonic developmental transition. The review addresses the functions and interactions of thyroid hormone and corticosteroid signaling in amphibian development (metamorphosis) as well as the developmental roles of these two pathways in vertebrate evolution.

## 1. Introduction

**Figure 1:**
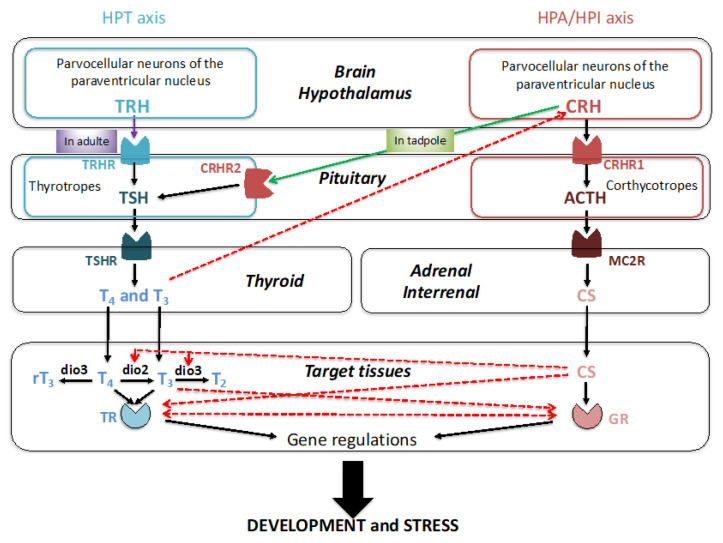
Hypothalamus–pituitary–thyroid and hypothalamus–pituitary–interrenal neuroendocrine axes and their interactions. The main actors of the hypothalamic–pituitary–thyroid (HPT) axis are in blue. Abbreviations: dio, deiodinase; T_4_, thyroxine; T_3_, tri-iodothyronine; rT_3_, reverse triiodothyronine; T_2_, diiodothyronine; TR, thyroid hormone receptor; TRH, thyrotropin releasing hormone; TRHR, thyrotropin releasing hormone receptor; TSH, thyrotropin; TSHR, thyrotropin receptor. The action of TRH on TSH in amphibians is observed only in juvenile and adult frogs (purple arrow). In tadpoles, TSH is controlled by CRH (green arrow). The main actors of the corticotropic (hypothalamic–pituitary–adrenal/interrenal, HPA/HPI) axis are in Bordeaux. Abbreviations: ACTH, adrenocorticotropin; CRH, corticotropin-releasing hormone; CRHR1, corticotropin-releasing hormone receptor 1; CRHR2, corticotropin-releasing hormone receptor 2; GR, glucocorticoid receptor; MC2R, melanocortin receptor 2.

Thyroid hormones (THs) play essential roles in vertebrate growth and development by controlling cell differentiation, migration, and homeostasis. The hypothalamic–pituitary–thyroid (HPT) is the neuroendocrine axis (Figure 1, Blue color) composed of brain thyrotropin-releasing hormone (TRH) neurons, pituitary thyrotropes (cells producing thyroid stimulating hormone or TSH), and the thyroid gland [1]. TRH acts via its receptor expressed by the pituitary thyrotropes to stimulate the synthesis of pituitary TSH. Following its release, TSH binds its receptor expressed on the thyroid gland to induce the production and release of THs, mainly thyroxine (T_4_) and to a lesser extent triiodothyronine (T_3_). T_4_, often considered to be the precursor for T_3_, is converted to T_3_ by monodeiodinases at target tissues. Both THs bind to TH nuclear receptors (TRs) in various tissues. Finally, THs negatively feedback on the brain (hypothalamic TRH neurons, thyrotropes).

Corticosteroids (CSs) are stress hormones controlling development, cellular energy metabolism, skeletal growth, cardiac functions, immune functions, reproduction, and cognition. The hypothalamic–pituitary–interrenal axis (HPI) in amphibians is the neuroendocrine axis (Figure 1, Bordeaux color) where the neurohormone, corticotropin-releasing hormone (CRH), controls at the pituitary level the production and release of corticotropin (adrenocorticotropic hormone, ACTH) [1]. ACTH leads to production and release of CSs from the adrenal cortex in amniotes or interrenal cells in fish and amphibians. CSs can act on various tissues via two receptors, mineralocorticoid receptor (MR) and glucocorticoid receptor (GR). Similar to THs, CSs negatively feedback on the brain (hypothalamic CRH neurons, corticotropes).

In some amphibians, the main action controlled by the HPT and HPI axes is the developmental body change corresponding to metamorphosis (from the Greek meta- (change) and morph (form)) [2]. This post-embryonic developmental process is strongly linked to a need to adapt to a change in habitat for complex life cycles with different ecophases. This period is also a developmental window sensitive to diverse biotic and abiotic factors that can induce an organismal response by modulating hormone production by the HPT and HPI neuroendocrine axes that then profoundly influence behavior, morphology, growth, and development [3]. Stressors, such as pond desiccation, predation, or limited food availability, can cause activation of the HPI axis [4]. In normal and stressed conditions, the synthesis and release of TSH is under the control of CRH in tadpoles [3,5] (Figure 1, Green arrow). The classical regulation of TSH by TRH seen in other vertebrates occurs only after metamorphosis [6] (Figure 1, purple arrow). CRH injection into tadpoles is able to accelerate metamorphosis by increasing TSH and ACTH levels [7,8]. Other interactions between the HPT and HPI axes can be involved in the control of the timing and the rate of metamorphosis (Figure 1, red arrows). CSs can repress the expression of inactivating deiodinases and promote the expression of activating deiodinases, thus contributing to T_3_ availability in target cells [9]. Further, TH can act in synergy with CSs at the level of their respective receptors. CSs increase the amount of TR transcripts [10] and the binding capacity of T_3_ in nucleus [11]. Conversely, T_3_ can increase the expression of GR in the tail and decrease its expression in the brain [12]. Finally, some genes are synergistically regulated by T_3_ and CSs during metamorphosis [13,14,15] and some through direct transcriptional regulation by TR and GR [16]. In the present review, we will highlight the hormonal control of metamorphic changes by THs, with increasing evidence pointing to prominent roles by CSs. We will also highlight the conservation during vertebrate evolution of both hormones signaling and their interaction.

## 2. T_3_ and TR in *Xenopus* Development

**Figure 2:**
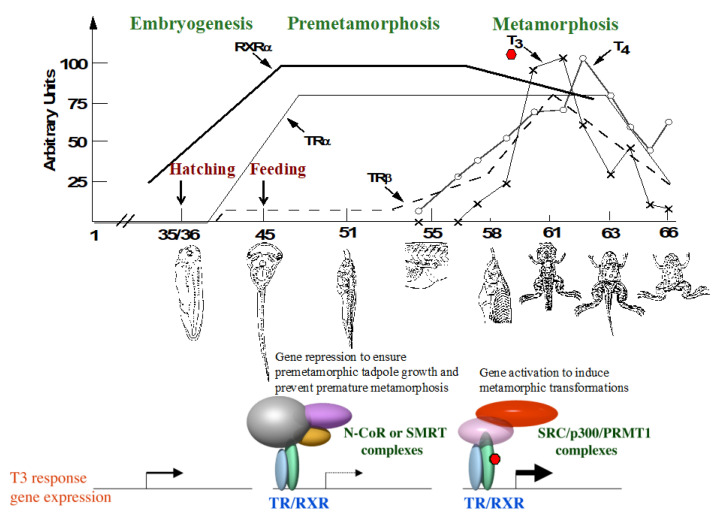
TR function during frog development. (**Top**). Correlations of plasma T_3_ and T_4_ and mRNA levels of TRα, TRβ, and RXRα with *Xenopus laevis* development. The embryos hatch around stage NF35, and a feeding stage tadpole is formed by stage NF45. Note that the expression of TRα (solid line) and RXRα (bold line) is activated to high levels when tadpole feeding begins, while high levels of T_3_/T_4_ and TRβ mRNA (broken line) are present only during metamorphosis. (**Bottom**). Regulation of T_3_-inducible genes in frog development. During embryogenesis, there is little T_3_/T_4_ or TR expression, and TR target genes are expressed at basal levels independent of TRs or T_3_/T_4_. During premetamorphosis between stage NF45 but before stage NF54 when there is little T_3_/T_4_ present, the expression of TRs, mainly TRα and RXRs, causes repression of the genes due to the binding of the TR target genes by unliganded TR/RXR heterodimers that recruit corepressor complexes. The synthesis of endogenous T_3_/T_4_ after stage NF54 leads to formation of liganded TRs that recruit coactivator complexes for the activation of target genes and metamorphosis.

Over a century ago, Gudernatsch discovered that one or more substances in the thyroid could accelerate anuran metamorphosis [17]. Subsequent studies have shown that T_3_ is the causative agent of amphibian metamorphosis [2,17,18,19,20]. First, plasma concentrations of T_3_ and T_4_ in the anuran *Xenopus laevis* correlate with metamorphosis [21] (Figure 2). Second, blocking the synthesis of endogenous T_3_ prevents natural metamorphosis, leading to the formation of giant tadpoles that remain as tadpoles well past the time needed to complete natural metamorphosis but will resume metamorphosis once T_3_ is available [18]. Third, T_3_ treatment of premetamorphic tadpoles as early as at the onset of feeding leads to precocious metamorphosis [2,18,22]. Finally, T_3_ can even induce metamorphic transformations in organ cultures of premetamorphic tadpoles [23,24,25], suggesting that individual organs autonomously undergo T_3_-dependent, organ-specific metamorphosis. This section on THs reviews the evidence for the importance of THs in *Xenopus* metamorphosis.

### 2.1. Establishing a Dual Function Model for TR during Development

T_3_ functions mainly by binding to its TRs to regulate target gene transcription, although T_3_ can also bind to cytoplasmic and plasma membrane proteins to exert non-genomic effects on cells, at least in cultured mammalian cells [26], although no non-genomic roles have been studied in amphibians [27,28,29,30,31,32,33,34]. There are two highly conserved TR subtypes, encoded by *TRα* and *TR*β genes, respectively, in vertebrates [28]. There is only a single gene for each TR subtype in the diploid anuran *Xenopus tropicalis,* although the pseudo-tetraploid anuran *Xenopus laevis* has two duplicated *TRα* and *TR*β genes [35,36,37].

Extensive biochemical and molecular studies have shown that for T_3_-induced genes, TRs function mainly as heterodimers with 9-cis retinoic acid receptors (RXRs) and constitutively bind to T_3_ response elements (TREs) in target genes [28,30,31,38,39]. They repress the target genes in the absence of T_3_ but activate them when T_3_ is present by recruiting corepressor complexes and coactivator complexes, respectively, to target promoters [40,41,42,43,44,45]. These T_3_-dependent dual effects of TR on transcription, coupled with the developmental expression profiles of the TR genes in *Xenopus laevis* [35,46,47,48,49], have led to the proposal of a dual function model for TR during Xenopus development (Figure 2) [50]. According to this model, T_3_-inducible genes are expressed at basal levels during embryogenesis when there is little TR or T_3_ present. By the end of embryogenesis when a feeding tadpole is formed (stage NF45 according to the *Xenopus* developmental table, [51]), the expression of these genes is no longer needed and is thus repressed, in part due to the activation of the expression of *TR* and *RXR* genes, especially *TRα* and *RXRα*. As there is little T_3_ present in premetamorphic tadpoles, TR/RXR heterodimers binds to TREs in these target genes to recruit corepressors and repress their expression. Such gene repression helps to prevent premature initiation of metamorphosis and allows the tadpole to grow sufficiently before metamorphosis. When the endogenous T_3_ level rises after stage NF54 due to the activation of T_3_ synthesis and secretion, T_3_ binds to TRs, and the resulting liganded TR/RXR heterodimers in turn recruit coactivator complexes to activate the expression of target genes, leading to metamorphosis. While this model was proposed largely based on available information for *Xenopus laevis*, it is likely applicable toward at least other anurans, particularly the diploid *Xenopus tropicalis*, which is highly related to the pseudo-tetraploid *Xenopus laevis*, due to the conservation in TR function and temporal expression profiles of the TR genes [37].

Many published studies have provided strong support for this model of TR function during development. First, transient and transgenic overexpression of wild-type and mutant TRs in *Xenopus laevis* have shown that TR is both necessary and sufficient to mediate the metamorphic effects of T_3_ [52,53,54,55,56,57,58,59]. More recently, with the development of gene editing technologies, both *TRα* and *TR*β genes have been knocked out individually or together in the diploid *Xenopus tropicalis* [60,61,62,63,64,65,66,67,68,69,70,71,72,73,74]. Consistent with the model and the lack of TR expression during embryogenesis, knockout of either *TRα* or *TR*β or both *TR* genes has little effect on embryogenesis (Table 1). As expected from the fact that there are only two *TR* genes in *Xenopus tropicalis*, knocking out both *TR*α and *TR*β abolishes premetamorphic tadpole responses to exogenous T_3_ treatment [71]. Importantly, *TR* double-knockout animals initiate metamorphosis precociously compared to the wild-type siblings, i.e., they reach the onset of metamorphosis (stage NF54) at a younger age (Table 1) [71]. Thus, unliganded TR controls metamorphic timing, as predicted from the dual function model. Once metamorphosis begins, the *TR* double-knockout tadpoles develop more slowly, taking a much longer time to develop from stage NF54 to stage NF58 (prometamorphosis) or from stage NF58 to stage NF61 (early metamorphic climax) (Table 1) [71]. These findings indicate that liganded TR regulates the rate of metamorphic progression. The *TR* double-knockout tadpoles are subsequently stalled at stage NF61 for up to 2 weeks before eventual death. Thus, liganded TR is not only important for a proper rate of metamorphic progression but is also essential for the completion of metamorphosis and tadpole survival (Table 1) [71], supporting the dual function model.

Mechanistically, chromatin immunoprecipitation (ChIP) assays have shown that TRs bind to TREs in target genes constitutively, both in premetamorphic tadpoles when there is little T_3_ and during metamorphosis when T_3_ levels are high [75]. Furthermore, as predicted by the model, unliganded TR in premetamorphic tadpoles recruits corepressor complexes, and disrupting corepressor complex recruitment via transgenic expression of a dominant negative form of the corepressor N-CoR that can bind to unliganded TR but cannot form a functional corepressor complex leads to derepression of T_3_-target genes in premetamorphic tadpoles and premature initiation of metamorphosis in *Xenopus laevis* [57,76,77,78]. Thus, unliganded TR recruits corepressor complexes to repress gene expression to control metamorphic timing. ChIP assays have also shown that coactivators, such as the histone acetyltransferases SRC3 and p300 and the arginine methyltransferase PRMT1, are recruited to T_3_ target genes during metamorphosis when T_3_ is present [79,80,81,82,83]. More importantly, transgenic expression of a dominant negative SRC3 or p300 that disrupts coactivator complex formation in *Xenopus laevis* or knocking out *SRC3* in *Xenopus tropicalis* inhibits T_3_ signaling and metamorphic progression [79,81,82,84], demonstrating an important role of coactivator complex recruitment by liganded TR for metamorphosis.

### 2.2. Temporal Correlation of TR Subtype Expression with Organ-Specific Metamorphosis

**Figure 3:**
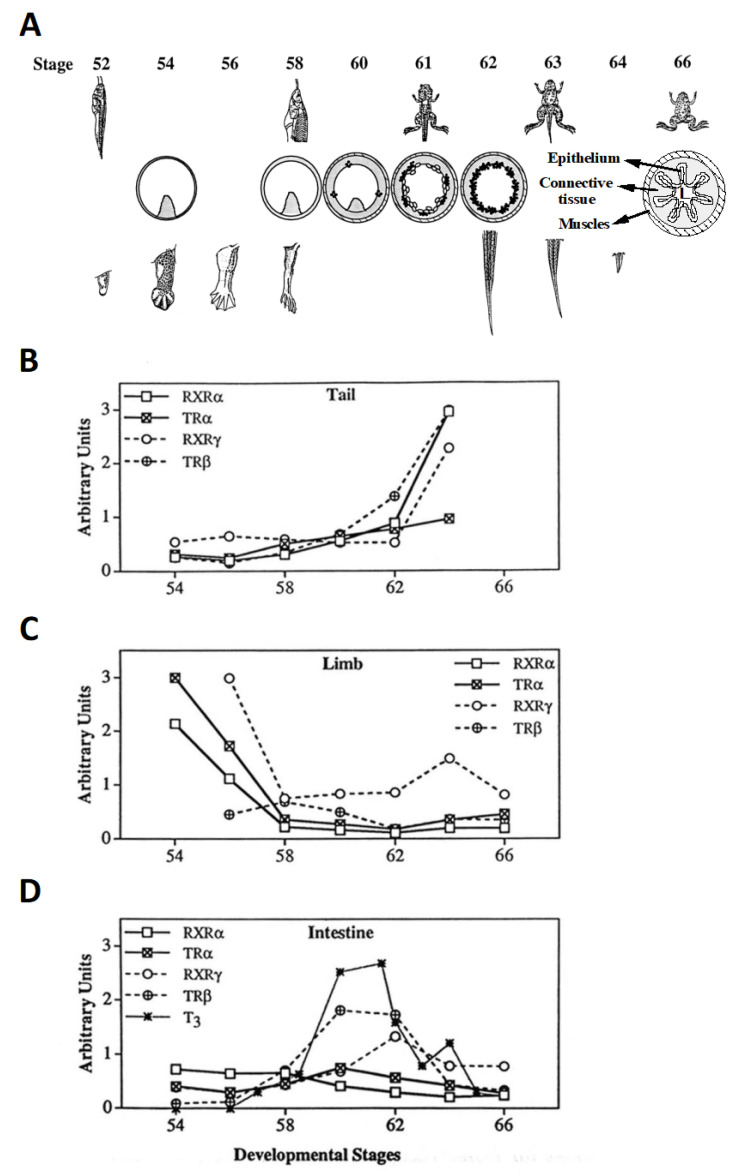
The expression of TR and RXR genes temporally correlates with organ-specific metamorphosis. (**A**). Temporal regulation of metamorphic organ transformations in *Xenopus laevis*. Tadpole developmental stages and ages are based on [51]. The tails at stages NF62-NF66 are drawn to the same scale to show resorption (no tail is left by stage NF66), while the tadpoles, intestinal cross-sections (stages NF54-NF66), and the hindlimbs (stages NF52-NF58) at different stages are not to scale in order to show stage-dependent morphological changes during metamorphosis. Tadpole small intestines have a single epithelial fold, where connective tissue is abundant, while frogs have a multiple-folded intestinal epithelium, with thick connective tissue and muscles. Black dots, proliferating adult intestinal epithelial cells; circles, apoptotic larval intestinal epithelial cells; L, intestinal lumen. (**B**–**D**). TR-subtype-dependent temporal regulation of TR and RXR expression in the hindlimb, intestine, and tail. Note that in general, the mRNA levels are high when the organs undergo metamorphosis. During tail resorption (stages NF62-NF66), TRβ expression is strongly upregulated, while TRα expression is only moderately increased (**B**). In contrast, during limb morphogenesis (stages NF54-NF58), TRα mRNA level is high, while TRβ is low (**C**). During intestinal remodeling, TRβ expression is strongly upregulated, while TRα mRNA level is slightly increased (**D**). Adapted from [27,49].

Anuran metamorphosis transforms essentially every organ/tissue in a tadpole. However, different organs undergo distinct changes at different developmental stages. Temporal coordination of the changes in different organs are essential to ensure the formation of a frog and survival of the animal. For example, while treatment of tadpoles with low levels of exogenous T_3_ can induce premature metamorphosis or accelerate metamorphosis, T_3_, even at levels similar to peak levels at the climax of metamorphosis, can cause lethality when added to rearing water of premetamorphic tadpoles, likely due to discoordinated metamorphic changes in different organs. The limbs, intestine, and the tail represent three types of organs with distinct metamorphic changes at different developmental stages (Figure 3A) [2,51]. The limbs are adult-specific organs and are formed early during metamorphosis through de novo growth and differentiation. The initiation of digit formation in the hindlimbs at stage NF54 is considered at the onset of metamorphosis, and limb morphogenesis is largely complete by stage NF58, the beginning of metamorphic climax. The tail, however, is the last organ to complete metamorphosis, with tail length reduction taking place after stage NF61/NF62 and completion of tail resorption through cell death by the end of metamorphosis at stage NF66. The intestine remodels in between limb development and tail resorption, involving both degeneration of the larval epithelium through programmed cell death and de novo formation of adult epithelial stem cells, followed by their proliferation and differentiation.

The mechanism for the temporal regulation of metamorphosis of different organs is not fully understood. There is a TR subtype-dependent temporal correlation of TR expression with organ specific changes, at least in the limb, tail, and intestine in both *Xenopus laevis* and *Xenopus tropicalis* (Figure 3B) [37,49]. In the hindlimbs, the TRα mRNA level is high, while TRβ is low during limb morphogenesis (stages NF54-NF58). However, in the tail, the expression of both *TR* genes is low prior to any significant tail length reduction before stage NF60. During tail resorption (stages NF60-NF66), TRα expression is moderately increased, while TRβ expression is strongly upregulated, especially during stages NF62-NF64 when rapid tail length reduction occurs. Similarly, during intestinal remodeling, mainly between stages NF58-NF66, TRβ expression is strongly upregulated while TRα expression is increased slightly. Furthermore, there is also a temporal correlation, although somewhat weaker, of RXR expression with metamorphosis in these three organs (Figure 3B). Thus, TR may have subtype-dependent roles in the temporal control of organ-specific metamorphosis, most likely as TR/RXR heterodimers.

Recent gene knockout studies have provided genetic support for the subtype-specific roles of TR in different organs. Consistent with the early and high levels of TRα but not TRβ expression in the hindlimb during development, *TRα* but not *TR*β knockout leads to premature initiation of metamorphosis, i.e., accelerated limb development (note that limb morphology is typically used to stage tadpoles from stages NF50-NF58 [51]) (Figure 2 and Figure 3, Table 1) [60,61,62,63,64,65,70]. Limb development is similarly accelerated in premetamorphic *TRα* knockout and *TR* double-knockout tadpoles, suggesting that TRβ does not play any significant role in regulating limb development during premetamorphosis. Furthermore, during metamorphosis between stages NF54-NF58 when the T_3_ plasma level rises, *TR*α knockout delays limb development, while *TR*β knockout has little effect. Limb development between stages NF54-NF58 is further delayed in *TR* double-knockout animals compared to *TRα* knockout ones (Table 1), suggesting that liganded TRβ also contributes to limb development during this period. As limb development is essentially complete by stage NF58, there are no detectible effects of any *TR* knockout on the limb after stage NF58.

The subtype-specific effects of *TR* knockout on the tail are the opposite of those for the limb, but again agree with the temporal expression profiles of the *TR* genes (Figure 3 and Table 1). Here, *TRα* knockout has little effect on tail resorption, which occurs at metamorphic climax stages (Table 1) [60,61,63,65], while *TR*β knockout delays tail resorption, particularly the notochord, (Table 1) [62,63], likely due to the much more dramatic increase in TRβ, especially in the notochord, but not TRα expression in the tail during tail resorption (Figure 3) [69]. Tadpoles lacking both TRα and TRβ can develop up to stage NF61 but are then stalled for up to two weeks before death without any significantly shortening of the tail (Table 1) [71], suggesting that both TRα and TRβ contribute to tail resorption and can compensate for each other to different degrees.

A different picture emerges for the intestine, where knockout of either *TRα* or *TR*β delays intestinal remodeling (Figure 2) [60,61,62,63,64,65,70], although TRβ is upregulated much more dramatically than TRα (Figure 2). Since the absolute levels of TRs, especially TR proteins, are unknown, it is possible that TRα and TRβ are expressed at comparable levels during intestinal remodeling despite the difference in their developmental regulation patterns. Furthermore, *TR* double knockout has much more severe effects on intestinal remodeling, preventing both larval epithelial cell death and adult intestinal stem cell development [71,85], supporting important but compensatory roles of TRα and TRβ in the intestine.

### 2.3. TR Is Essential for Completion of Metamorphosis but Derepression of Gene Expression due to TR Double-knockout Allows for Precocious Development of Many Adult Organs

While knockout of *TRα* and *TR*β individually leads to significant developmental changes in different organs/tissues, it does not prevent the completion of metamorphosis nor the formation of reproductive adults, which have normal external morphology, indicating that TRα and TRβ can compensate for the loss of each other to ensure completion of development, although with some alternations in developmental rate and timing in different organs. However, when both *TR* genes are knocked out, the tadpoles are developmentally stalled at the climax stage NF61 before eventual death (Table 1), while even the presence of a single copy of wild-type *TR*β gene allows for development to reproductive adults [71]. Many adult organs/tissues are formed by stage NF61 in the *TR* double-knockout animals [71,74,85]. This contrasts with tadpoles devoid of T_3_, which do not develop past stages NF51/NF52 [2,18]. Thus, the derepression of TR target genes due to the double-knockout appears to be sufficient for the formation of many adult organs/tissues.

The most noticeable effect of *TR* double-knockout is the lack of tail resorption as the knockout animals are stalled at stage NF61 for up to two weeks before death, a period longer than the one week or so that is needed for the wild-type tadpoles at stage NF61 to complete tail resorption (Table 1) [71]. The resorption of the gills, which are also larval specific organs, and the degeneration of the larval intestinal epithelium are also inhibited in the *TR* double-knockout tadpoles [71,85]. Thus, it is likely that the formation of most adult organs/tissues merely require the derepression of TR target genes (although additional activation by liganded TR may speed up the process), while larval tissue degeneration requires liganded TR. The failure of the larval tissue degeneration in *TR* double-knockout tadpoles may contribute to their lethality at the climax of metamorphosis.

## 3. Corticosteroids (CSs) and Their Receptors in *Xenopus* Development

More than twenty-five years after it was discovered that a substance from the thyroid gland induces metamorphosis [17], treatment of tadpoles with adrenal steroids was found not only to increase the rate of metamorphosis induced by T_3_, but also to have no developmental effect in the absence of T_3_ except to prevent or delay the onset of metamorphosis [86,87,88,89]. These foundational observations reflect the two accepted developmental roles of CSs in frog metamorphosis: (1) direct actions on tissues to increase their sensitivity/responsivity to T_3_ [10,90] and (2) direct and perhaps indirect action on the HPI/HPT axes to influence plasma levels of TH and CSs [91]. By increasing tissue sensitivity/responsivity to T_3_, CSs accelerate T_3_-dependent organ transformations, and by influencing the shape of the developmental profile of plasma hormone levels, CSs regulate the initiation and overall rate of metamorphosis. Compared to TH, it has been difficult to study CSs in metamorphosis because surgical removal of interrenal tissue has not been successful [92]. In addition, corticosteroid synthesis inhibitors have not been able to overcome the counteracting effect of the HPI axis to partially restore the tissue content of CSs [4,93]. Recent gene disruption studies have shed new light on CSs and their receptors in development [94,95]. This section on CSs will review the evidence for the importance of CSs in *Xenopus* metamorphosis.

### 3.1. Production of CSs during Development

The predominant CSs synthesized in tadpole interrenal cells are corticosterone (CORT, a glucocorticoid) and aldosterone (ALDO, a mineralocorticoid), and some cortisol is also made in some species [96]. An important role in natural metamorphosis for CSs was first indicated by histological studies showing increased interrenal cell activity during metamorphosis [97,98]. Later, the output of hormones by the interrenals was quantified by radioimmunoassay showing CORT and ALDO plasma levels peak at the climax of metamorphosis [14]. The synthesis of both CORT and ALDO in interrenal tissue is stimulated by the pituitary hormone adrenocorticotropin (ACTH) encoded by the gene *pomc* (CRH) [91]. The stimulation of ALDO synthesis by the renin–angiotensin system known in frogs and other vertebrates has not been established in tadpoles [99]. All known effects of exogenous CSs in development can be accomplished by both CORT and ALDO or related steroids and agonists, such as dexamethasone, desoxycortiocsterone, and hydrocortisone [14]. CSs have several known physiological roles in tadpoles in addition to affecting development, including carbohydrate and lipid catabolism, immune function, and morphological and behavioral response to predators [100,101,102]. CSs are also known to regulate salt/water homeostasis in adults, but no such action has been observed in tadpoles until adult skin starts to form during metamorphosis [92]. The presence of two CSs, CORT and ALDO, with likely distinct physiological and perhaps developmental roles in tadpoles, complicates the analysis of the endogenous actions of how these two CSs may regulate development.

### 3.2. Nuclear Receptors for Corticosteroid Signaling during Development

CSs function by binding to nuclear receptors in target cells to regulate transcription, and cell surface receptors for CSs can carry out non-genomic effects as well [103,104]. There are two nuclear receptors for CSs, the glucocorticoid receptor (GR) and the mineralocorticoid receptor (MR), present in all vertebrates [105]. Both GR and MR have nearly universal expression among tissues in the adult (Xenbase) and are both expressed in all tadpole tissues examined [106,107]. GR and MR are sequestered in the cytoplasm in the absence of ligand and thus, unlike TH receptors, do not regulate gene expression unless ligand is present [104]. MR binds both CORT and ALDO with equal and high affinity, whereas GR binds only CORT and at a lower affinity compared to CORT/MR binding [108]. At supraphysiological concentrations, ALDO can activate GR, which has confounded efforts to identify distinct developmental roles for GR versus MR [107,108].

### 3.3. Requirement of CSs for Metamorphosis

The fact that CSs per se have no known developmental role suggests the possibility that metamorphosis does not require signaling by CSs, where T_3_-dependent metamorphosis could still occur but without the modulation in rate. Early experiments with receptor antagonists and steroidogenic enzyme inhibitors supported this suggestion. The inhibitors, metyrapone and amphenone B, delayed but did not prohibit T_3_-induced metamorphosis in vivo [4,93,109]. Similarly, treatment of tadpoles with the GR receptor antagonist RU486 in vivo delayed but did not prevent survival through metamorphosis [102]. However, neither of these experimental approaches rule out a requirement for CSs because inhibition of corticosteroid signaling was incomplete. In addition, in-vivo T_3_ treatment alone without CORT often causes death, but this death is attributed to dysregulated organ transformations rather than lack of the presence of CSs. Further, T_3_ treatment in culture allowed for complete tail resorption in the absence of CSs [108,110,111], indicating that CORT is not necessary, but the tail tip results do not necessarily reflect the in vivo situation. Thus, the above experiments (1) do not give definitive insight into the need for CSs in metamorphosis, (2) do not rule out if CSs have developmental effects independent of T_3_, and (3) give little insight into which corticosteroid (CORT versus ALDO) or which receptor (GR versus MR) may influence metamorphic events.

**Figure 4:**
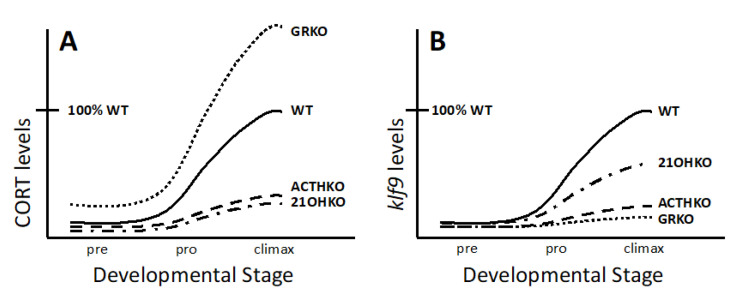
Levels of plasma CORT and *klf9* expression in corticosteroid mutant frogs. (**A**). Compared to wild-type (WT) tadpoles, glucocorticoid receptor knockout (GRKO) tadpoles have high corticosterone (CORT) levels from lack of negative feedback, adrenocorticotropic hormone knockout (ACTHKO) tadpoles have low CORT levels from lack of adrenal stimulation by ACTH, and *cyp21a2*, encoding 21-hydroxylase, knockout (21OHKO) tadpoles have low CORT levels from lack of a steroidogenic enzyme to make CORT. (**B**). The expression of *klf9* (Krüppel-like factor 9) is directly induced by CORT and by T_3_. The expression of *klf9* is also indirectly increased by CORT via CORT’s action to increase tissue responsivity to T_3_. Thus, compared to WT tadpoles, 21OHKO tadpoles have reduced *klf9* levels from reduced CORT signaling caused by lack of CORT but abundant CORT synthesis precursors able to bind CORT receptors with lower affinity. ACTHKO and GRKO have low *klf9* levels from minimal to no contribution of CORT/GR signaling to induce *klf9* via CORT signaling per se or via an increase in tissue responsivity to T_3_.

Disruption of genes involved in corticosteroid production and signaling are starting to shed new light on the essential components and their basic roles during metamorphosis (Table 2). In contrast to prior expectations, *pomc* mutants, which lack ACTH to stimulate interrrenal production of CSs, die at NF64 (near the end of metamorphosis halfway through tail resorption) [94]. In addition, these mutants did not show a significant peak of CORT at NF 62 (metamorphic climax) and could be rescued to survive to adulthood by continuous exogenous CORT treatment (Figure 4A). Similarly, GR mutants die but at an earlier stage (NF61-62), likely because lack of GR signaling is more severe than in ACTH mutants where basal levels of CORT are still present to provide some corticosteroid signaling [95]. These mutants show that corticosteroid signaling through GR is required for survival through metamorphosis.

Gene disruption was also carried out for *cyp21a2* to more fully eliminate CSs compared to ACTH mutants (unpublished data). The gene *cyp21a2* encodes 21-hydroxylase, which functions at the end steps in CORT and ALDO synthesis. Surprisingly, *cyp21a2* mutants survived metamorphosis, despite having low plasma CORT levels, similar to ACTH mutants (Figure 4A). The persistent, although low, CORT levels in *cyp21a2* mutants were accompanied by high levels of steroid synthesis precursors or intermediates. To explain *cyp21a2* survival, *cyp21a2* mutants had, unlike in ACTH mutants, maximal stimulus for adrenal steroid production by virtue of lack of negative feedback, allowing for high ACTH levels to stimulate the production of high levels of CORT and ALDO precursors. Despite their lower affinity, the agonist activity of such precursors at heightened concentrations may signal enough through GR and MR for survival through metamorphosis. These results from *cyp21a2* mutants are consistent with results from GR and ACTH mutants, indicating that a sufficient level of corticosteroid signaling through GR is required for survival through metamorphosis.

### 3.4. Necessity of Corticosteroid-Mediated Enhancement of Thyroid Hormone Signaling

The cause of death at metamorphic climax in GR and ACTH mutant animals is not precisely known. In these mutants, TH signaling was impaired, as indicated by reduced expression of T_3_-response genes, likely due to loss of corticosteroid signaling that enhances tissue sensitivity and responsivity to T_3_ in wild-type animals (Figure 4B) [9,10,94,95]. Importantly, both mutants can be rescued from death and allowed to proceed completely through metamorphosis if treated with exogenous T_3_ [94,112]. Survival of GR and ACTH mutants through metamorphosis by use of exogenous T_3_ alone suggests that these mutants had insufficient TH signaling to progress beyond metamorphic climax. These results support that endogenous corticosteroid signaling in wild-type animals is required to achieve sufficient T_3_ signaling in end-stage metamorphic events that need the highest amount of T_3_ signaling. This result with GR and ACTH mutants is consistent with the death at metamorphic climax in TR mutant tadpoles, which are also not able to achieve metamorphic events that need high T_3_ signaling. Sufficient T_3_ signaling requires high enough expression of TR in combination with high levels of plasma and intracellular T_3_, none of which obtain in ACTH, GR, or TRα/β double knockouts.

### 3.5. Developmental Roles of Corticosteroids Independent of TH

Frog GR and ACTH mutants seem to die from reduced T_3_ signaling rather than from lack of a vital action of CSs on development [94,95]. In contrast, mice mutant for GR or MR die at birth and weaning from impaired lung development and salt wasting, respectively, presumably due to direct actions of CSs on these processes [113]. Nevertheless, rescue of GR and ACTH mutant tadpoles with exogenous T_3_ does not rule out the possibility that corticosteroid signaling may be vital for development independent of T_3_ action. In the case of ACTH mutants, basal levels of CORT and perhaps ALDO were present to activate the wild-type GR and/or MR enough to stimulate a putative vital role. Similarly, wild-type MR was present in GR mutants to conduct possible vital roles. For both GR and ACTH mutants, the animals died before the end of metamorphosis, precluding observation of a potential vital role of corticosteroid signaling during that period. Future studies with MR mutants and double GR/MR mutants are required to rule out vital actions of CSs through these receptors. Similarly, *cyp11a1* mutants (P450scc, required for synthesis of all steroid hormones from cholesterol) are required to rule out vital roles of CSs independent of T_3_. In light of the critical interactions between CSs and T_3_ identified in amphibian development in combination with the conserved nature of hormonal control of development among vertebrates detailed in the next section, the vital actions attributed solely to CSs, e.g., the role of CSs in mammalian lung development may have heretofore undocumented critical involvement of T_3_.

## 4. Conservation during Vertebrate Evolution of Thyroid Hormone and Corticosteroid Action in Post-Embryonic Development

**Figure 5:**
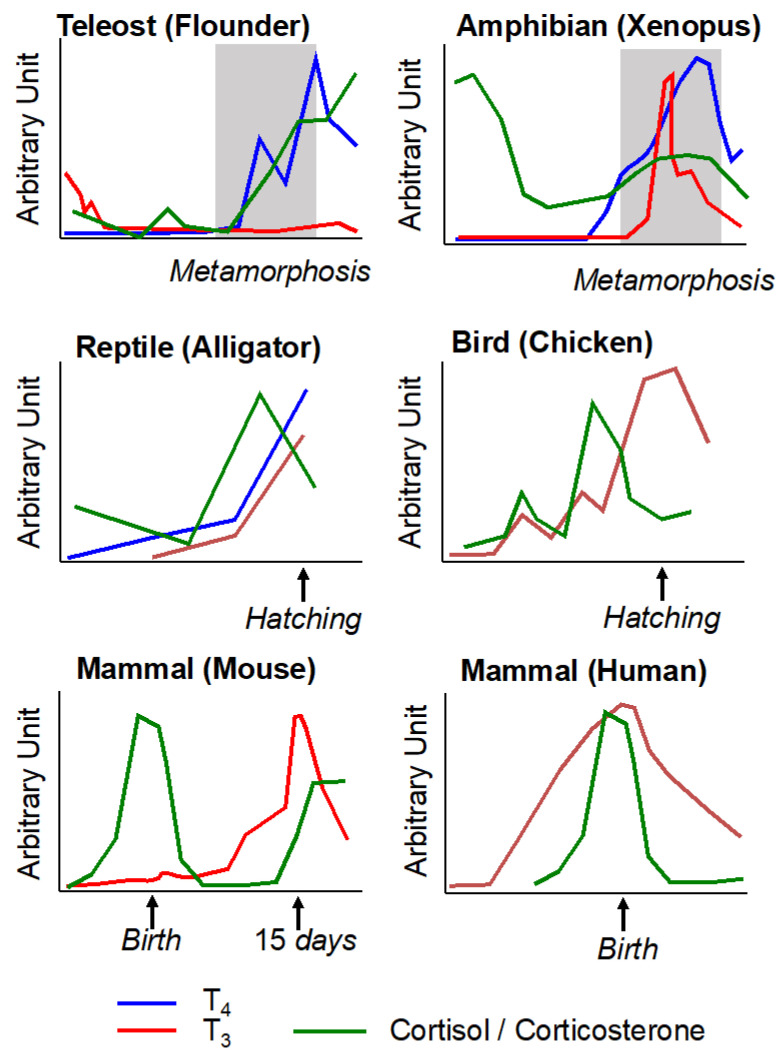
Schematic representation of changes in baseline thyroid hormones and corticosteroids during post-embryonic development. Blue line—T_4_, thyroxine; red line—T_3_, triiodothyronine; green line—cortisol/corticosterone, corticosteroids. Thyroid hormone levels are from flounder (teleost fish, [115]), Xenopus (amphibian, [21]), alligator (reptile, [116]), chicken (bird, [117]), and mammals (mouse, [118] and human, [20]). Corticosteroid concentrations are from Wada 2008 [114].

Metamorphosis is an emblematic type of post-embryonic developmental process. This life stage transition from one ecological niche to a different ecological niche is also present in various metazoan phylogenetic groups. Here, we will present the contributions of TH and CSs in metamorphosis and developmental transitions in other vertebrates, including larval and secondary metamorphosis in teleost fishes, egg hatching in sauropsids, and birth in mammals. Two neuroendocrine axes, the hypothalamic–pituitary–thyroid and the hypothalamic–pituitary–adrenal (mammals and sauropsids)/interrenal (amphibians and teleost fishes) axes, are central players in the regulation of these life transitions. To highlight the evolutionary conservation of these two axes, all vertebrates have both axes (Figure 1) with a peak of T_3_ and a peak of CSs that co-occur during post-embryonic development (Figure 5). In addition, similar T_3_ and CS concentration profiles are observed in certain birds and reptiles at hatching and during molting, as well as in certain teleost fishes at hatching and during larval metamorphosis and smoltification [114].

### 4.1. Metamorphosis in Teleost Fishes

T_3_ has an important role in fish egg hatching [119], but it will not be considered in this review. Several studies also report developmental changes controlled by T_3_ during larval to juvenile transition in various teleosts such as grouper *Epinephelus coioides* [120], seabream [121], gobiid *Sicyopterus lagocephalus* [122] or clownfish *Amphiprion percula* [123]. Our review will focus on the two types of metamorphosis involving morphological and physiological modifications leading to niche/habitat changes. One occurs at the larval to juvenile transition in elopomorphs and pleuronectiforms, and the second one occurs in juveniles of some diadromic migratory teleosts, i.e., smoltification.

Metamorphosis of the flatfish represents the classical larval metamorphosis in teleosts, with spectacular morphological changes such as the migration of one eye to the opposite side of the head [124]. These changes are also accompanied by physiological (such as development of gastrointestinal tract to adapt to the novel food resources in a new habitat) and behavioral (pelagic to benthic life and locomotion) changes. Larval tissue T_4_ and T_3_ concentrations peak at metamorphic climax in the Japanese flounder *Paralichthys olivaceus* [125], in spotted halibut, *Verasper variegatus* [126], Atlantic halibut *Hippoglossus hippoglossus* [127], and summer flounder, *Paralichthys dentatus* [128]. T_3_ is involved in many metamorphic changes, including shortening of fin rays, eye migration, and asymmetry [124]. Treatments of flatfish with T_3_ or anti-thyroid drugs lead respectively to acceleration or delay of these classical changes [129,130,131,132]. The developmental profiles of deiodinases, which convert T_4_ to T_3_ or inactivate T_3_, coincide with the rise of TH levels. During sole metamorphosis, DIO2 activity, which converts T_4_ to T_3_, increases during metamorphosis, while DIO3 activity, which inactivates T_3_, declines at the mid-late metamorphic period [133]. As during amphibian metamorphosis, the expression of *TR* increases at metamorphic climax [134,135].

As seen in amphibians, CS concentration also reaches a peak at climax in Japanese flounder [115]. CS treatment of larval fishes does not trigger the changes observed during metamorphosis, as in eye migration and settling (benthic) behavior, but it does induce shortening of the second fin ray [130]. Furthermore, in spotted halibut, CS treatment increases the development of adult-type pigment cells [136]. With respect to interactions between T_3_ and CS signaling, CS treatment leads to enhanced hepatic and renal DIO2 activities in juvenile Senegalese sole [137], but this CS effect on extrathyroidal T_3_ production has not yet been confirmed during larval metamorphosis. In the Japanese flounder, a permissive effect of CSs on T_3_ action is observed in vitro but not in vivo during metamorphosis [130]. Thus, future studies are required to investigate the potential CS/T_3_ interactions in teleost metamorphoses as observed in amphibians.

A secondary metamorphosis in teleosts is exemplified by smoltification in salmonids, which allows for the transition from freshwater to the ocean. Smoltification involves morphological changes, such as body silvering and fin darkening, as well as behavioral changes, with swimming activity in open space and migration, and finally physiological changes related to adaptation to seawater and imprinting [138,139]. Many parallel features exist between flatfish metamorphosis and salmonid smoltification [140], the first one being the control by T_3_. Here again, plasma T_4_ and/or T_3_ levels rise at the time of smoltification [140]. Manipulation of T_3_ levels supports a major role of T_3_ in adaptation to a new environment, such as change in visual pigments, olfaction, metabolism, swimming behavior, and migration [139,141]. A peak of expression of *tshβ1b* was recently measured at the time of initiation of the downstream migration in Atlantic salmon [142]. In all the tissues involved in smoltification-related changes, expression of TR has been detected [143,144,145,146] but does not change between the different stages [146]. The HPI axis is also involved during smoltification with a peak in plasma cortisol levels [147,148,149,150]. Manipulation of the HPI axis can affect pigmentation [151] and osmoregulation [152]. The involvement of GR was supported by data showing a stimulatory effect of cortisol in gill tissue blocked by the GR antagonist, mifepristone [153] and an increase in GR concentration and *gr* expression [154,155,156,157]. Few studies report interactions between T_3_ and CS signaling during smoltification. T_3_ can increase CRH neurogenesis [158], but CS treatment can lower plasma T_3_ and reduce the increases of TR mRNA and protein expression [159,160], suggesting some antagonistic/negative effects of CSs on T_3_ action.

### 4.2. Egg Hatching in Sauropsids

Hatching in birds and reptiles is characterized by a transition from an aqueous environment to a terrestrial environment. For birds, it is important to differentiate between precocial and altricial species [161,162]. In precocial species where hatchlings are immediately mobile and independent after hatching, plasma T_3_ concentrations of the embryos increase during the peri-hatch period [161]. In contrast, hatchling altricial birds need to be fed and thermoregulated by their parents in the nest and have low circulating concentrations of T_3_ that increase only after hatching [163,164]. Manipulation of egg T_3_ levels influences avian hatching time [165,166,167]. T_3_ availability is controlled by deiodinase activity in chicken with high levels of DIO3 during embryogenesis [117,168,169,170] and DIO1, another enzyme that can convert T_4_ to T_3_, whereas DIO2 levels increase to a peak around hatching [117,169,170]. T_3_ effects are driven by TR expressed in many embryonic tissues as observed in chicken [171,172].

Around the time of hatching, an increase in avian embryonic CSs is observed in plasma [173,174,175,176]. Injection of CORT in the turkey or chicken yolk sac can increase the incubation period when injected at early stages of embryonic development, but injection of CORT in the same species can shorten incubation period when injected at late stages [175,177,178]. CSs act on chicken lung prior to hatching to stimulate surfactant synthesis [179]. The CS effects in chicken are mediated by GR expressed during the pre-hatching period [176]. Interactions between T_3_ and CS signaling have also been observed in birds. Again, in chicken embryos, hypothyroidism leads to medullar hypertrophy in the adrenal gland [180], and CS or ACTH injection leads to increased plasma T_3_ concentrations via increased DIO2 activity [181] and decreased DIO3 activity [182].

In reptiles, T_3_ signaling is associated with hatching, as exemplified by an increase in T_3_ levels in saltwater crocodile *Crocodylus porosus*, [116] and with thyroid exhibiting the features of a fully active gland in the grass snake *Natrix natrix* [183]. In turtle, injection of an antithyroid drug delays hatching time [184], and treatment with T_3_ shortens incubation duration [185]. T_3_ treatment also increases DIO1 and DIO2 activity in crocodile [116] as well as the secretion of pulmonary surfactant in crocodile [186] and turtle [187]. CSs also stimulate pulmonary surfactant in turtle [187], crocodile [186] and lizard [188]. An increase in CSs is observed around hatching in alligator [189], lizard [190], and turtle [191], but a decrease is observed in crocodile [116]. In lizard, CS injection accelerates egg hatching [192]. In alligator, GR expression during embryonic development suggests a potential role for CSs in tissue maturation before hatching [193]. Finally, note that both T_3_ and CSs are involved in the regulation of surfactant production in reptiles, similar to that observed in other amniotes [194].

### 4.3. Birth in Mammals

During mammalian development, T_3_ availability is tightly controlled by developmental changes in endogenous synthesis in the thyroid and tissue deiodinase activity, leading to a rise in plasma T_3_ concentration near term [195] or shortly after delivery [118,196,197]. T_3_ has a major regulatory role on fetal growth and development [195], including maturation of the central nervous system [198], maturation of the respiratory system including synthesis of the components of surfactant [199,200,201], maturation of the skeleton [202], and maturation of the gastro-intestinal tract [203]. These post-embryonic developmental maturations are crucial to cope with the change of environment (from aquatic to terrestrial) and of nutrition (from parenteral to enteral). An increase in plasma fetal CSs is detected near parturition in many mammals [196,204,205,206]. Two striking examples of exogenous CS effects are: first, the injection of ACTH or CSs into the ovine fetus leads to premature delivery [207,208], and second, CSs are routinely injected to favor lung maturation in human preterm birth [209]. As observed for T_3_ signaling, many roles of CSs on organ maturation, including brain, lung, digestive tract, and skeleton, have been demonstrated and are the subjects of recent reviews [210]. Before birth, T_3_ and CSs synergize for the maturation of these organs. This synergy is partly the result of CS-induced increases in circulating T_3_ and local T_3_ bioavailability [211,212].

## 5. Conclusions

In all of the developmental transitions described here, a common involvement of HPT and HPA/HPI axes is observed, including the cross-talk between these two (Figure 1). The hormone-dependent developmental processes are diverse with morphological, behavioral, and physiological changes essential to adapt to a new environment and/or new way of life. The conservation along vertebrate evolution of the mechanisms underlying these major biological processes is striking from fish and amphibian metamorphoses to egg hatching in sauropsids and birth in mammals. The synchronized increase in T_3_ and CS production and release, coupled with the local control of hormone availability and expression of receptors, allow tissues to fine-tune the two hormone responses. The multiple cross-talks between the HPT and HPA/HPI axes represent critical mechanisms by which vertebrates modulate their perinatal/post-embryonic development as well as their responses to a changing environment. Of all these interactions, one differs between mammals and non-mammalian vertebrates. In amphibian, sauropsids, and teleost fishes, the CRH appears to be a coordinator of activation of both HPT and HPA/HPI axes at the time of developmental transitions involving stimulation of TSH production and release. It is not the case in mammals, where TRH and CRH respectively control the HPT and HPA axes early during development. Mechanisms of integration of environmental and internal cues, and regulation/operation of these neuroendocrine axes represent key questions in an “eco–evo–devo” perspective of metamorphosis and more generally post-embryonic developmental processes. The roles played by developmental transitions in the innovation, adaptation, and plasticity of life cycles throughout vertebrates are then essential, especially within the current context of global anthropogenic disturbance. The impact of environmental factors, such as global warming, habitat destruction, and endocrine disruptors on the HPT and HPA/HPI axes, and regulation of developmental transitions must be seen as a threat for biodiversity. Humans are not spared. In preterm birth, which is the leading cause of perinatal morbidity and mortality in developed countries, the affected infants often experience HPT and HPA dysfunctions [213,214,215]. Antenatal CS administration reduces the complications after preterm birth, while T_3_ supplementation has not been shown to be beneficial. However, the benefit of a therapy is still a controversy [209]. Thus, key questions regarding HPT and HPA/HPI functions during post-embryonic developmental transition still remain to be answered to protect biodiversity and human health.

## Figures and Tables

**Table 1:** Organ-dependent effects in single and double TRα/β knockout *Xenopus tropicalis*.

		Embryogenesis	Premetamorphosis	Prometamorphosis	Early Metamorphic Climax	Late Climax
		Stages 1–45	45–54	54–58	58–62	62–66
**Rate of development**	TRα KO	No effect ^1^	Acceleration	Delay	No effect ^1^	No effect ^1^
	TRβ KO	No effect ^1^	No effect ^1^	No effect ^1^	Minor delay	Delay
	TRα + β KO	No effect ^1^	Acceleration	Further Delay	Delay, then death	N/A ^2^
**Limb**	TRα KO	No effect ^1^	Acceleration	Delay	No effect ^1^	No effect ^1^
	TRβ KO	No effect ^1^	No effect ^1^	No effect ^1^	No effect ^1^	No effect ^1^
	TRα + β KO	No effect ^1^	Acceleration	Further Delay	No effect ^1^	N/A ^2^
**Intestine**	TRα KO	No effect ^1^	No effect ^1^	No effect ^1^	Delay	Delay
	TRβ KO	No effect ^1^	No effect ^1^	No effect ^1^	Delay	Delay
	TRα + β KO	No effect ^1^	No effect ^1^	No effect ^1^	Complex effects ^3^	N/A ^2^
**Tail**	TRα KO	No effect ^1^	No effect ^1^	No effect ^1^	No effect ^1^	No effect ^1^
	TRβ KO	No effect ^1^	No effect ^1^	No effect ^1^	Delay	Delay ^4^
	TRα + β KO	No effect ^1^	No effect ^1^	No effect ^1^	Inhibition	N/A ^2^

^1.^ “No effect” refers to little or no obvious effect. ^2.^ Development is stalled at stage 61 for up to 2 weeks or so before eventual death, while wild-type tadpoles begin tail resorption around stage 61 and complete the process in a week or so. ^3.^ Inhibition of both larval epithelial cell death and adult epithelial stem cell development, but premature formation of adult epithelial folds. ^4.^ Especially notochord.

**Table 2:** Effects of corticosteroid mutants on hormone signaling.

	ACTH KO	GR KO	cyp21a2 KO
**GR**	Expressed	Absent	Expressed
**MR**	Expressed	Expressed	Expressed
**CORT**	low	high	low
**ALDO**	Not measured	high	Not measured
**steroid precursors**	Not measured	high	high
**CORT responsivity**	Not measured	Absent	Not measured
**TH responsivity**	very low	very low	low
**development rate**	slow	fast then slow	slow
**survival**	no	no	yes
